# Decapitation Experiments Combined with the Transcriptome Analysis Reveal the Mechanism of High Temperature on Chrysanthemum Axillary Bud Formation

**DOI:** 10.3390/ijms22189704

**Published:** 2021-09-08

**Authors:** Yujie Yang, Sagheer Ahmad, Qingqing Yang, Cunquan Yuan, Qixiang Zhang

**Affiliations:** 1 Beijing Key Laboratory of Ornamental Plants Germplasm Innovation & Molecular Breeding, Beijing 100083, China; yujieyang@bjfu.edu.cn (Y.Y.); sagheerhortii@gmail.com (S.A.); h2o_yqq@bjfu.edu.cn (Q.Y.); 2 National Engineering Research Center for Floriculture, Beijing 100083, China; 3 Beijing Laboratory of Urban and Rural Ecological Environment, Beijing 100083, China; 4 Engineering Research Center of Landscape Environment of Ministry of Education, Beijing 100083, China; 5 Key Laboratory of Genetics and Breeding in Forest Trees and Ornamental Plants of Ministry of Education, Beijing 100083, China; 6 School of Landscape Architecture, Beijing Forestry University, Beijing 100083, China

**Keywords:** chrysanthemum, high temperature, WGCNA, double decapitation, hormonal regulation

## Abstract

Temperature is an important factor that largely affects the patterns of shoot branching in plants. However, the effect and mechanism of temperature on axillary bud development in chrysanthemum remains poorly defined. The purpose of the present study is to investigate the effect of high temperature on the axillary bud growth and the mechanism of axillary bud formation in chrysanthemum. Decapitation experiments combined with the transcriptome analysis were designed. Results showed that the axillary bud length was significantly inhibited by high temperature. Decapitation of primary shoot (primary decapitation) resulted in slower growth of axillary buds (secondary buds) under 35 °C. However, secondary decapitation resulted in complete arrest of tertiary buds at high temperature. These results demonstrated that high temperature not only inhibited axillary bud formation but also retarded bud outgrowth in chrysanthemum. Comparative transcriptome suggested differentially expressed gene sets and identified important modules associated with bud formation. This research helped to elucidate the regulatory mechanism of high temperature on axillary bud growth, especially bud formation in chrysanthemum. Meanwhile, in-depth studies of this imperative temperature signaling can offer the likelihood of vital future applications in chrysanthemum breeding and branching control.

## 1. Introduction

Heat stress causes morpho-physiological changes and influence plant growth and development [1]. Temperature is an important extrinsic cue that integrates with the growth and development of lateral branches. Previous studies have shown that high temperature inhibits the differentiation of axillary meristem into axillary bud [2,3]. High temperature affects axillary meristem development in chrysanthemum ‘Improved Mefo’ and retards lateral bud formation in the new nodes of less-branching Chrysanthemum ‘Fucashi’ [4,5]. Histological examination of axillary buds after exposure to 33 °C day/27 °C night reveals premature differentiation of axillary meristematic tissue into non-meristematic parenchyma tissue [5]. Moreover, the lateral buds show disordered parenchymatous cells at high temperature (33 °C day/28 °C) [4]. Some researchers think that high temperature cannot affect the axillary meristem initiation; rather it affects the development and formation of axillary bud [3]. The above discussion indicates that high temperature can affect the chrysanthemum axillary bud formation from the perspective of phenotype and anatomy. However, the detailed mechanism is largely unknown.

Phytohormones respond to changes caused by abiotic stresses, such as high temperature as the increase in temperature modulates hormone levels [1]. Hormones play significant roles in the development of axillary buds in leaf axils. Auxin and cytokinin are two important hormones which are closely related to axillary bud formation. In addition, gibberellin has also been reported to involve the regulation of axillary bud initiation [6]. Low concentration of auxin in the leaf axils drives axillary meristem initiation in Arabidopsis [7,8]. Contrarily, cytokinin promotes the formation of axillary meristem in Arabidopsis [9]. Auxin, cytokinins and strigolactone are the key internal factors that control the axillary bud outgrowth. In these hormones, auxin has been pivotal to theories about apical dominance, a process wherein growing tip decides the fate of axillary buds. The canalization based auxin transport model states that production of auxin (indole-3-acetic acid) in shoot tip and its descending flow barricades the lateral bud outgrowth [10,11]. In the second messenger model, cytokinin synthesis is negatively regulated by the auxin flowing in the main stem [12,13], which induces bud outgrowth; while the strigolactone levels are positively regulated [14], that inhibits bud outgrowth [15,16]. BRANCHED1 (BRC1) is a transcription factor which normally expresses in seized axillary buds and in mutants that show profound branching. It significantly integrates the antagonistic effect of cytokinins and strigolactone [16,17,18]. It has also been reported that abscisic acid (ABA) abundance in axillary bud is directly related to the degree of dormancy of axillary buds [19,20,21,22]. Recently, the studies on the relationship between sucrose and lateral branch development have aroused great interest [23]. In fact, the post-decapitation pea plant exhibited initiation of bud outgrowth before the auxin content changes in the adjacent stem tissues [24]. It is an admitted fact that bud outgrowth innervates plenty of sugar metabolic activities and sugar transportation within buds [25,26,27,28,29].

Chrysanthemum is the second most important floriculture crop, after rose at top, in the worldwide floriculture trade [30,31]. In single stem cut chrysanthemum, the growth of axillary bud is the most disliked trait. The conventional approach of manual disbudding to obtain single stem flower is damaging, labour-intensive and consumes one-third of cultivation expenditures [32]. Lateral branch development generally consists of two stages. In the first stage, axillary meristem initiates in the leaf axil and forms axillary bud, which is called axillary bud formation. In the second stage, the axillary bud outgrows and develops into lateral branch, which is called axillary bud outgrowth [33,34]. These processes are affected by intrinsic and extrinsic stimuli.

With the above in mind, previous findings have suggested the significance of temperature as a stimulator of bud formation at morphological and anatomical levels in chrysanthemum and concluded that high temperature mainly affects axillary bud formation. However, the detailed mechanism is largely unknown. Therefore, this study reveals the influence of high temperature on the whole process of chrysanthemum lateral branch development and the molecular mechanism of chrysanthemum bud formation in response to temperature. We found that high temperature not only inhibited axillary bud formation but also affected axillary bud outgrowth. Decapitation experiments combined with the comparative transcriptome analysis further revealed that high temperature acts as a modulator to control bud formation by integrating the network formed by hormones inside the bud. In addition, amounts of differentially expressed gene sets and important modules associated with bud formation were identified. Our study, thus, provides a solid foundation to analyze the regulation of axillary bud growth in chrysanthemum in response to high temperature. On the basis of possessing fundamental role in signal transduction in plants, in-depth studies of this imperative temperature signaling can offer the likelihood of vital future applications in chrysanthemum breeding and branching control.

## 2. Results

### 2.1. High Temperature Affects Axillary Bud Formation and Bud Outgrowth

Traditional studies suggest that the effect of high temperature on shoot branching is mainly reflected in the inhibition of axillary meristem differentiation to form axillary buds at the axil.

**Figure 1:**
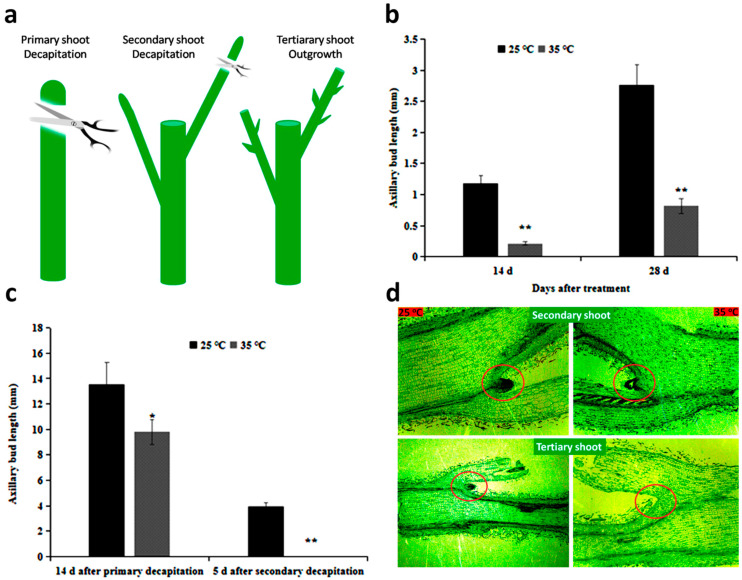
Axillary bud growth status in contrasting temperatures. (**a**) Models of decapitation experiments. Decapitation means removing upper 3 cm portion of main shoot including shoot apex of plants. The first decapitation performed on the un-decapitated plants was regarded as primary decapitation and the axillary buds/shoots that emerged at the first axil after primary decapitation were regarded as secondary buds/shoots. Decapitation that was done on the newly formed first axillary shoots after primary decapitation was regarded as secondary decapitation. Axillary buds/shoots formed after secondary decapitation were regarded as tertiary buds/shoots; (**b**) The length of axillary buds under normal (25 °C) and high temperature (35 °C) treatment without decapitation; (**c**) The length of axillary buds under normal (25 °C) and high temperature (35 °C) treatment with decapitation; (**d**) Paraffin slicing micro-observation of axillary buds after primary decapitation and secondary decapitation. The red circle position represents the axillary bud in first axil position. Significance is shown at * *p* < 0.05 and ** *p* < 0.01 level.

To reveal the effect of high temperature on the whole process of chrysanthemum lateral branch development, we first designed two growth environments (day and night temperature of 35/25 °C was regarded as high temperature regime and day and night temperature of 25/15 °C was regarded as normal temperature regime) and examined the bud length in response to each temperature treatment. Results showed that the axillary bud length was significantly shorter in high temperature environment than in normal temperature regime 14 d and 28 d after treatment, suggesting that high temperature inhibited axillary bud growth (Figure 1b).

To reveal the detailed effect of high temperature on the whole process of chrysanthemum axillary bud development, a secondary decapitation experiment was designed and the bud response was examined (Figure 1a). Results showed that the axillary buds especially those at the upper axis position emerged quickly after primary decapitation in both temperature treatments. However, compared with the normal temperature, axillary bud length in high temperature was significantly shorter (Figure 1c). Histological results supported that axillary buds at the upper axis position had complete formation and outgrowth after primary decapitation (Figure 1d). These results indicated that the restriction of bud length by high temperature at this stage was mainly manifested as the suppression of axillary bud outgrowth. After secondary decapitation, buds at the upper axis position emerged quickly in normal temperature treatment, however, no signs of growth were observed for axillary bud in high temperature (Figure 1c). Microscopic observation also demonstrated that axillary bud was not formed in high temperature (Figure 1d). These results indicated that high temperature inhibited axillary bud formation. Combining with the primary decapitation and secondary decapitation results, we concluded that high temperature not only inhibited axillary bud formation but also suppressed bud outgrowth.

### 2.2. Comparative Analysis of Differentially Expressed Genes (DEGs) during Axillary Bud Formation at 25 °C and 35 °C Conditions

**Figure 2:**
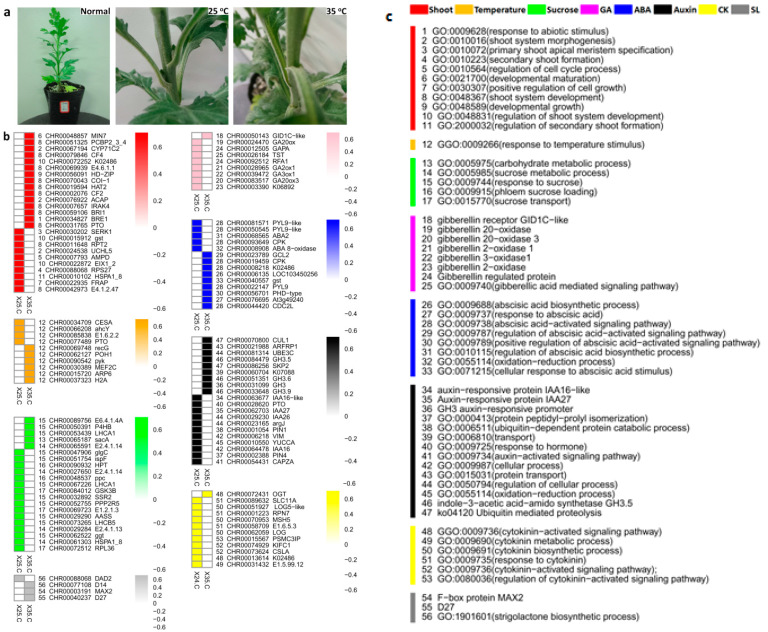
Bud outgrowth pattern and antagonistic expression of specific DEGs in non-decapitated plants. (**a**) Non-decapitated plants growing in 25 °C and 35 °C regimes. Top one shows the normal growth of plant before temperature treatment. The lower two plants show the effect of temperature on bud outgrowth. Bud outgrowth is suppressed in under the influence of 35 °C. This is the core idea to go for transcriptomic analysis and find some hormonal and genetic regulators of bud arrest due to increase in temperature; (**b**) Contrasting expression of DEGs expressed for pivotal regulators of bud formation and outgrowth; (**c**) explanation of colors and details of numbers as biological process annotations used in ‘b’.

A total of 1024469496 raw reads were generated and mapped to chrysanthemum genome (v2.0) using TopHat. A detailed comparative analysis of the DEGs was conducted to reveal the important integrators of axillary bud formation at contrasting temperature regimes. Significantly enriched DEGs were found for shoot formation, temperature stimulus, sucrose homeostasis and transport and hormonal regulators, including ABA, gibberellic acid (GA), auxins, cytokinins and strigolactones (Figure 2). Plenty of important biological processes were annotated to DEGs related to shoot formation, including secondary shoot formation system, developmental growth, primary shoot apical meristem specification and regulation of cell cycle. For temperature, all the DEGs were annotated to temperature stimulus. Sucrose transport and phloem sucrose loading were the important biological processes regarding sucrose homeostasis. Moreover, major plant hormone biosynthesis and signalling related DEGs were observed with completely opposite expression intensities at contrasting temperatures (Figure 2). It can be seen that ABA and GA show antagonistic behavior in contrasting temperatures in perspective of bud formation. Similarly, cytokinins and auxins depicted opposite responses in both temperatures. Strigolactone is always abundant at high temperatures, suggesting important roles in bud check.

### 2.3. Weighted Gene Co-Expression Network Analysis (WGCNA) for Sucrose Homeostasis and Bud Growth Dynamics

**Figure 3:**
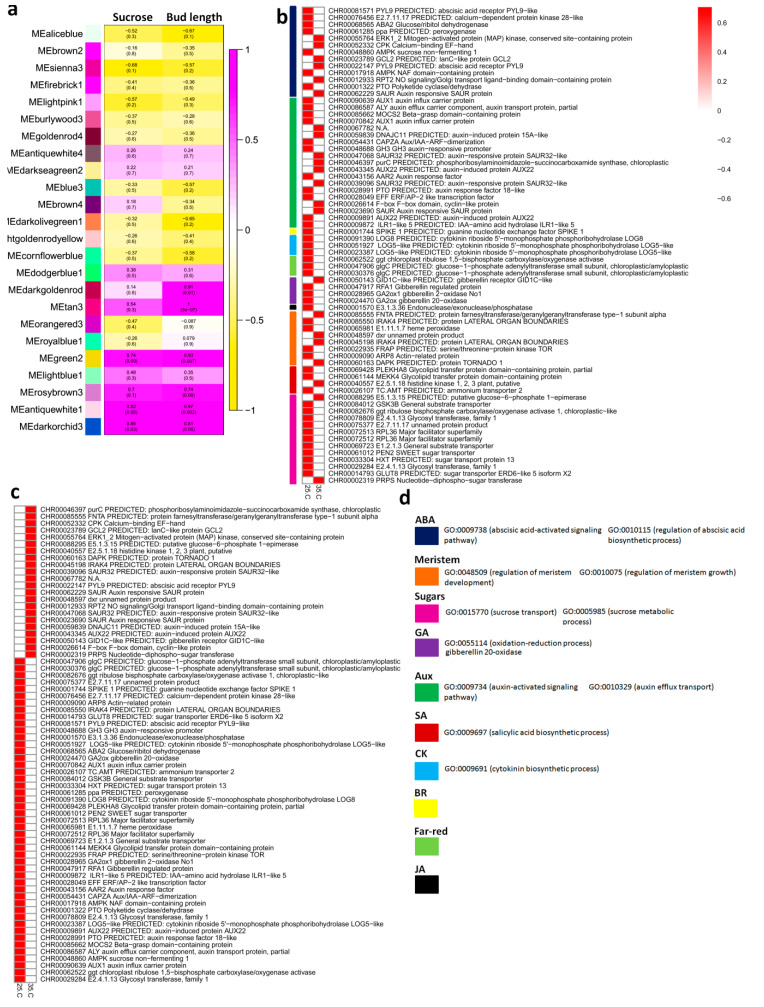
WGCNA analysis of DEGs. (**a**) Module enrichment for sucrose homeostasis and bud dynamics. MEdarkorchid3 is the most enriched module for sucrose signalling and bud outgrowth; (**b**) Enrichment of important terms in the module MEdarkorchid3; (**c**) Clustering of terms enriched in MEdarkorchid3 for important regulators of bud dynamics as shown in part ‘b’; (**d**) Biological process annotations of some important bud regulators.

WGCNA was performed to identify regulatory modules for sucrose stimulation and bud growth dynamics (Figure 3). Among the significantly enriched modules, darkorchid3 contained the most significantly enriched terms with contrasting expression intensities at 25 °C and 35 °C (Figure 3b). A number of DEGs were found related to important regulators of bud dynamics, including meristem development (GO:0048509), ABA biosynthesis (GO:0010115), sucrose transport (GO:0015770), gibberellin biosynthesis (GO:0055114), auxin efflux transport (GO:0010329) and cytokinin biosynthesis (GO:0009691). Clustering analysis of darkorchid3 showed that most of the genes were upregulated at 25 °C as compared to 35 °C (Figure 3c).

### 2.4. The Expression of Hormone-Related Core Genes Affected by High Temperature

**Figure 4:**
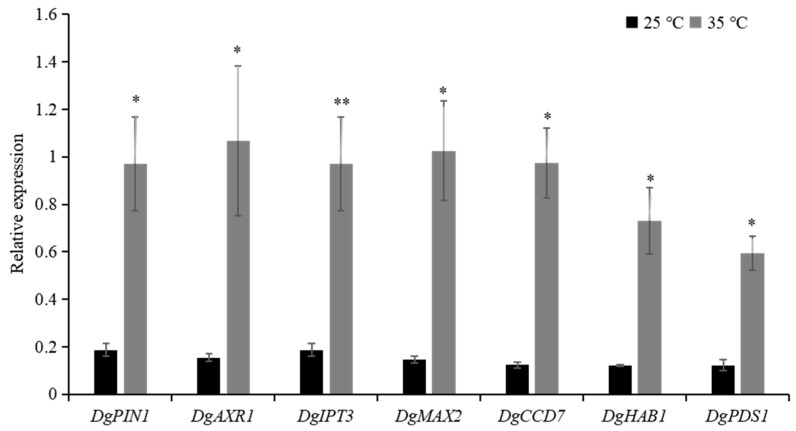
Expression of hormone-related genes in axillary buds 12h after secondary decapitation (bud formation) and non-decapitation under 25 °C and 35 °C conditions. Auxin transport gene *DgPIN1*, auxin signalling gene *DgAXR1*, cytokinin synthesis gene *DgIPT3*, SL signal transduction gene *DgMAX2,* SL biosynthesis gene *DgCCD7*, ABA core signal transduction gene *DgHAB1* and ABA responsive gene *DgPDS1*.Data are mean ±SE of three biological replicates. Asterisks indicate significant differences between treatment conditions 25 °C and 35 °C at * *p* < 0.05 and ** *p* < 0.01 level.

To further reveal the involvement of hormones in the regulation of chrysanthemum bud formation, the expression of core hormone-related genes were examined by qRT-PCR after secondary decapitation. AUXIN RESISTANT 1 (AXR1) is an auxin signalling gene that is required to construct an F-box complex for ubiquitination of auxin repressors [35,36,37], resulting in commencement of auxin response genes [38]. It was shown to be responsible for axillary bud inhibition induced by auxin in Arabidopsis [39] and is also required to up-regulate strigolactone biosynthesis and to down-regulate cytokinin biosynthesis [15]. PIN-FORMED are the principal efflux carrier proteins controlling intercellular auxin transport [40]. The expression of *DgARX1* was increased in secondary decapitated buds in high temperature as compared to 25 °C (Figure 4), suggesting that high temperature stimulated auxin signalling, and thereby inhibiting bud development. *DgPIN1* transcripts, auxin efflux carrier analogue of *AtPIN1*, showed rapid accumulation in 12 h in high temperature buds (Figure 4), as compared to those in normal temperature. Increased expression of *DgPIN1* in high temperature may suggest that ability of auxin export from bud was enhanced.

ISOPENTENYL TRANFERASE (IPT) family catalyzes the rate-limiting step of cytokinin biosynthesis [41,42]. The expression of *DgIPT3* showed rapid increase in response to high temperature treatment after 12 h (Figure 4), suggesting that cytokinin-biosynthesis was promoted in axillary bud.

*MORE AXILLARY GROWTH (MAX)* genes are central to shoot branching regulation by strigolactone [35]. *DgMAX2* and *DgCCD7* are strigolactone signaling and strigolactone biosynthesis genes, respectively [43,44]. The expression of *DgMAX2* and *DgCCD7* were upregulated by high temperature treatment compared to control (Figure 4). It has long been reported that the content of ABA in axially buds is positively correlated with bud dormancy [19,20,22,45]. The expression of ABA signal transduction gene *DgHAB1* and ABA-responsive gene *DgPDS1* was significantly increased within 12 h in secondary-decapitated buds under high temperature (Figure 4), suggesting that ABA signal was enhanced in axillary buds in response to high temperature.

### 2.5. Hormonal Concentration Gradient and Axillary Bud Formation

**Figure 5:**
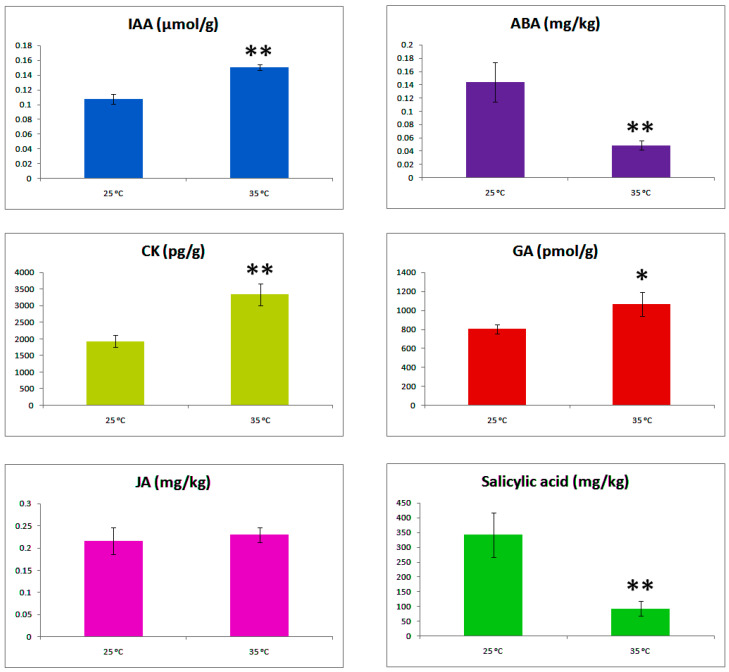
Concentrations of phytohormones in top axillary buds under 25 °C and 35 °C conditions during bud formation stage. IAA (indole acetic acid), CK (cytokinins), JA (jasmonic acid), ABA (abscisic acid), GA (gibberellic acid) and Sa (salicylic acid). Data are shown as mean ±SE of three biological replicates with forty-five axillary buds for each single repeat. Asterisks represent significant differences between the treatment conditions 25 °C and 35 °C at * *p* < 0.05 and ** *p* < 0.01 level.

Concentrations of six important hormones were ascertained for non-decapitated top axillary buds to know the initial involvement of phytohormones in axillary bud formation (Figure 5). Auxin and cytokinins showed synergistic relationship, both showing high concentrations at high temperature as compared to normal temperature. Interestingly, ABA and GA showed antagonistic relationship. ABA showed more concentration at normal temperature, while it was expected to show more at high temperature. Similarly, GA showed more concentration at high temperature. SA showed significant difference for high and normal temperatures. JA was not much affected by temperature change.

## 3. Discussion

The architecture of cut chrysanthemum is strongly influenced by its branching ability [46]. Therefore, shoot branching is an important ornamental trait that determines shape and quality of chrysanthemum [47]. Leaf axils introduce axillary meristems that form tiller buds that turn into shoots or remain dormant in unfavorable conditions [48]. Extensive research on bud outgrowth has evaluated a number of agents that support bud outgrowth such as sugars, cytokinins, and inhibitors such as ABA, auxin, and strigolactone [18,23,34,49,50]. However, little is documented on the influence of temperature on axillary bud burst in chrysanthemum. The current research focuses on temperature perception in the regulation of lateral branch development in cut chrysanthemum.

### 3.1. High Temperature Not Only Inhibited Axillary Bud Formation but Also Restricted Bud Outgrowth

Controlled climate is one of the most important strategies to control plant architecture. Temperature is an important agent that gains immense importance in plant kinetics and the fate of buds. Chrysanthemum (Powerhouse) produces few axillary shoots at 35 °C [51]. Moreover, high temperature hastens axillary bud burst in rose. We evaluated the effect of high temperature on the whole process of axillary bud development in chrysanthemum using decapitation experiments. The length of axillary buds under 35 °C was significantly shorter than axillary buds under 25 °C without decapitation, indicating that high temperature inhibits axillary bud development. Further studies revealed that axillary bud outgrowth rate was restricted under 35 °C after first decapitation. Interestingly, the axillary bud formation was completely obstructed under 35 °C after secondary decapitation (Figure 1). Therefore, high temperature not only inhibited axillary bud formation but also restricted bud outgrowth in chrysanthemum.

### 3.2. Temperature Makes a Way to Arrest Bud Burst through Hormonal Network

Previous studies have documented the role of cytokinins as stimulators of bud outgrowth in various species [12,13,52]. A number of phytohormones including salicylic acid (SA) and ABA increase their levels in high temperature, while others drop, such as GAs and cytokinins [53,54,55]. We report that high temperature up-regulates the biosynthesis of cytokinins in topmost axillary buds by up-regulating *DgIPT3*, a cytokinin biosynthesis gene (Figure 4). Strigolactones have recently been discovered as potential inhibitors of bud outgrowth and also as second messengers to auxin [14,56]. *CCD7* is a strigolactone biosynthesis gene [57]. In the current research high temperature upregulated *DgCCD7* within 12 h in tertiary buds (Figure 4). The expression of *DgMAX2*, a core signal transduction gene in strigolactone pathway, was significant upregulated within 12 h in tertiary buds (Figure 4), suggesting strong hold of high temperature on bud arrest through strigolactone control (Figure 3).

ABA is a general negative regulator of bud outgrowth. The expression of ABA signal transduction genes *DgHAB1* was increased within 12 h in primary and tertiary buds under high temperature and the expression of ABA-responsive gene *DgPDS1* was also significantly increased in response to high temperature (Figure 3). ABA levels and the related signaling pathways play significant role to suppress axillary bud outgrowth [58]. Abundant accumulation of ABA was reported to inhibit branches [20,58]. However, it is hard to decide how does temperature interacts with ABA to barricade axillary bud outgrowth and needs detailed study to precisely predict this interaction.

High availability of carbohydrates, such as glucose and sucrose, during high temperature represents a valuable physiological characteristic for tolerating heat stress [59]. Sucrose can manage heat stress through carbon allocation and signalling [60]. Despite being an important source of energy and carbon, sugars also possess hormone-like roles as key messengers during signal transduction [61]. Sucrose promotes the continuous synthesis of auxin inside buds and facilitates its export to the stem [23]. The role of sucrose in maintaining an auxin canal is strongly linked to *PIN1* regulation [62]. *AtPIN1*, *AtPIN2* and *AtPIN7* are positively regulated by sucrose [63,64]. WGCNA modules MEgreen2, MErosybrown3, MEantiquewhite1 and MEdarkorchid3 were enriched in sucrose signalling genes that may play crucial roles in bud regulation through hormonal pathways. Most of the sugar homeotic genes were upregulated in normal temperature buds as compared to high temperature (Figure 3b). Sucrose transport (GO:15770) and sucrose metabolic process (GO:0005985) were the major biological process shown by these DEGs (Figure 3d).

Major plant hormone biosynthesis and signalling related DEGs were observed with completely opposite expression intensities at contrasting temperatures (Figure 2b). In addition, a regulatory module significantly associated with sucrose stimulation and bud growth dynamics was identified by WGCNA. The modules with opposite expression genes were mainly concerned with meristem development, auxin efflux transport, ABA biosynthesis, cytokinin biosynthesis and gibberellin biosynthesis. Hormonal concentration gradient examination results showed that IAA, CK and GA levels were significantly increased under 35 °C conditions. Contrarily, ABA and SA levels were significantly decreased (Figure 5). These results suggested the involvement of hormone in regulating chrysanthemum axillary bud formation in response to high temperature.

Recent attempts have extensively evaluated the molecular mechanism underlying temperature signaling in plants. However, temperature control of bud arrest remains undefined, especially in chrysanthemum. Therefore, we demonstrated that temperature not only organizes early bud arrest but also controls the bud entrance into sustained outgrowth in chrysanthemum. Temperature-affected several hormonal entities involved in regulatory network of bud formation were identified, suggesting the significant role of temperature in specifying the bud formation. Therefore, studying the details of temperature regulation of axillary buds can do a great deal to comprehend the detailed mechanism of bud development in chrysanthemum.

## 4. Materials and Methods

### 4.1. Plant Material and Growth Conditions

Stem cuttings (*Chrysanthemum morifolium* ‘Jinba’) were grown in the greenhouse of Beijing Forestry University. Two month old seedlings at 15 axillary bud stage were transferred to controlled temperature chambers fitted with uniform light (Philips T8 TLD36/33 cold white tube, 120 µmol m^−2^ s^−1^ optical density). We designed two growth regimes. A day and night temperature of 35/25 °C was regarded as high temperature regime and a day and night temperature of 25/15 °C was regarded as normal temperature regime. Both growth environments were provided with equal light intensities and a day to night duration of 16/8 h.

### 4.2. Experimental Plan

The principle of the secondary decapitation experiment is that we create two independent processes, namely the axillary bud formation process and the axillary bud outgrowth process, and then evaluate the effect of temperature on each process. The specific operation process and principle was followed to perform this research. Firstly, plants were placed in a normal environment of 25 °C to ensure that the axillary buds had completely formed in the expanded leaf axils. At this time, the axillary buds were in the second stage of axillary development called axillary bud outgrowth. Then the plants were transferred to 25 °C and 35 °C environment respectively. Next, we decapitated the tip bud (namely primary shoot decapitation) to let the axillary bud outgrow quickly. At this time, the newly formed axillary buds were completely new, representing the stage of axillary bud formation. Then we re-decapitated the tip bud of secondary shoot (namely secondary shoot decapitation) to observe the growth status of axillary buds (namely tertiary buds). Our hypothesis is that if the growth of axillary buds shows differences between 25 °C and 35 °C environment after first decapitation, it indicates that temperature affects the bud outgrowth stage. If the growth of axillary buds shows differences between 25 °C and 35 °C environment after second decapitation, it indicates that temperature affects the bud formation stage.

Plants in each temperature environment were divided into two groups: (a) intact and (b) decapitation group. Plants in first group were not decapitated and regarded as control. After two weeks of growth, plants in second group were decapitated, removing upper 3 cm portion including apex. This decapitation was regarded as primary decapitation (Figure 1a). After two weeks of growth, decapitated plants in each temperature chamber were let to grow their axillary buds in the upper axis position. We regarded these newly outgrowing axillary buds as secondary axillary buds. Then the newly developed lateral branches at first axis position were further decapitated and this decapitation was called secondary decapitation and the buds appearing after secondary decapitation were called as tertiary buds (Figure 1a). After 12 h of each decapitation, the upper three buds and the lower three buds were sampled and immediately frozen in liquid nitrogen for further analysis. The length of non-decapitated axillary buds (control) was measured 14 d and 28 d after treatment. The length of secondary axillary buds was measured 14 d after primary decapitation. The length of tertiary buds was measured 5 d after secondary decapitation. The length was measured using digital vernier calipers. Samples and data were collected and recorded for 15 plants per treatment with 3 replicates.

### 4.3. Microscopic Documentation of Axillary Buds under Different Temperature Regimes

Stem cuttings containing single axillary buds were excised to see bud activities at micro level. The buds were fixed in FAA (formalin-acetic acid-alcohol) containing 70% ethanol, 37% formaldehyde acetic acid at a ratio of 18:1:1. Buds were then dehydrated using butyl alcohol series and embedded in paraffin. Embedded samples were cut into 10 µm thick strips using rotary microtone and then placed on microscopic slides. Slides were kept overnight at 40 °C and stained in Safranin-O and fast-green staining series [65] and were mounted using few drops of Permount medium (Fisher Scientific, Waltham, MA, USA). The slides were covered with cover glass and observed using a bright-field microscope.

### 4.4. Analysis of Hormones

Hormone examination sampling was conducted with the same procedure for *RNA-seq*. Major hormones related to bud formation were analysed with HPLC-MS/MS as described by Pan et al. [66].

### 4.5. RNA-Seq

90 plants were kept in high and normal temperature regime, respectively. The first expanded leaf was marked. After 22 days of growth, sampling was performed from top three axillary buds of expanded leaves. According to our pre-experiment, these three axillary buds were newly formed. Samples were collected for 15 plants per treatment with 3 replicates. The samples were collected in liquid nitrogen and stored at −80 °C until further use.

RNA-seq library was prepared from RNA isolated from axillary buds and sequencing was performed using HiSeq 2000 (Illumina platform). The sequence reads were aligned with the *Chrysanthemum nankingense* genome [67] using TopHat (v2.0.0) with default parameters. Differentially expressed genes between samples were determined at a false discovery rate of *p* < 0.05, and a fold change ≥2.

### 4.6. Weighted Gene Coexpression Network Analysis (WGCNA)

After RNA-seq, the WGCNA packages were used as previously reported [68,69]. The modules were generated specific to sucrose and bud length to decipher some important candidates for bud control.

### 4.7. Quantification of Gene Expression

Total RNA was extracted from frozen buds using MiniBESTplant RNA extraction kit (TaKaRa Biomedical Technology (Beijing) Co., Ltd, Beijing, China) including DNA removal as well, finally obtaining DNA-free RNA. The cDNA was obtained by performing reverse transcription of 1 µg RNA using PrimeScript^TM^RT reagent kit/system (TaKaRa Biomedical Technology (Beijing) Co., Ltd, Beijing, China). Quantitative real-time PCR (qRT-PCR) experiments were carried out with TaKaRaSYBR^®^*Premix Ex Taq^TM^*Π (TliRNaseH Plus) using a total mix of 15 µL including: 7.5 µL SYBR^®^*Premix Ex Taq^TM^*Π (TliRNaseH Plus)(2×), 0.6 µL forward primer (10 µM), 0.6 µL reverse primer (10 µM), 1.2 µL cDNA, 5.1 µL dH_2_O. CFX Connect Real-Time System was set to following programme: 30 s at 95 °C, then 40 cycles of 5 s at 95 °C and 30 s at 60 °C. Specific primers used for qRT-PCR are shown in Supplementary Table S1. Relative gene expressions were quantified using the expression of 18s as internal control.

### 4.8. Statistical Analysis

We performed one-way ANOVA in SPSS software (SPSS Inc., Chicago, IL, USA; ver. 16.0) to find statistical difference between two temperature regimes for axillary bud length, genes expression and hormones concentration. The significance is shown at * *p < 0.05* and ** *p < 0.01*.

## 5. Conclusions

Shoot branching is an important trait in breeding and production of chrysanthemum, especially for cut chrysanthemum. Meanwhile, high temperature is often encountered in the summer chrysanthemum production. However, the inherent mechanisms of influence of temperature on axillary bud development are largely unknown. This study demonstrates that high temperature not only inhibits axillary bud formation but also retards bud outgrowth in chrysanthemum. Comparative transcriptome analysis revealed amounts of differentially expressed gene sets and identified important modules associated with axillary bud formation. This study helps to elucidate the regulatory mechanism of high temperature on axillary bud growth especially for bud formation in chrysanthemum. On the basis of possessing fundamental role in signal transduction in plants, in-depth studies of this imperative temperature signaling can offer the likelihood of vital future applications in chrysanthemum breeding and branching control.

## Data Availability

The relevant data is available as supplementary material.

## Supplementary Materials

The following are available online at https://www.mdpi.com/article/10.3390/ijms22189704/s1.
