# Supplementary material for: Decapitation Experiments Combined with the Transcriptome Analysis Reveal the Mechanism of High Temperature on Chrysanthemum Axillary Bud Formation
Source: Int J Mol Sci. 2021 Sep 8;22(18):9704. doi: 10.3390/ijms22189704 (PMC8469267; doi:10.3390/ijms22189704)
Supplement: Supplementary file 1 [file ijms-22-09704-s001.zip › ijms-1349848-supplementary.pdf]

Supplementary Table S1. Primers used for quantitative real time PCR.

Gene	Primers
DgPIN1	Fwd: TGGCATTGCAACCAAGGATC
	Rev: AAATGGAAGCAGCAGCCATG
DgAXR1	Fwd: GGGCAAAACTGGAGAATTGA
	Rev: AGAAGATCGGCAGAGATCCA
DgIPT3	Fwd: TAAAGTAGTAGTCGTTATGGGTGCT
	Rev: CATCGTAAGAGAAGCCGTGC
DgMAX2	Fwd: GCACATACTGCACCATC
	Rev: GTAACGACAAACTCCTCTGG
DgCCD7	Fwd: TGGTGAAGTTCGATACTGTG
	Rev: CGTCGCTACCCTTTGATAC
DgHAB1	Fwd: TGGGCATCGTGTTTTTGGTG
	Rev: CATCACGTCCCATAACCCGT
DgPDS1	Fwd: ACCGGCATCAGCCAAATACT
	Rev: TTGACTATCCACGCCCTGAC
18S	Fwd: AAACGGCTACCACATCCAAG
	Rev: ACTCGAAAGAGCCCGGTATT
